# Impact of the estimated glomerular filtration rate on long-term mortality in patients with hypertensive crisis visiting the emergency department

**DOI:** 10.1371/journal.pone.0266317

**Published:** 2022-03-31

**Authors:** Byung Sik Kim, Mi-Yeon Yu, Hyun-Jin Kim, Jun Hyeok Lee, Jeong-Hun Shin, Jinho Shin

**Affiliations:** 1 Division of Cardiology, Department of Internal Medicine, Hanyang University College of Medicine, Hanyang University Guri Hospital, Guri, Republic of Korea; 2 Division of Nephrology, Department of Internal Medicine, Hanyang University College of Medicine, Hanyang University Guri Hospital, Guri, Republic of Korea; 3 Department of Biostatistics, Yonsei University Wonju College of Medicine, Wonju, Republic of Korea; 4 Division of Cardiology, Department of Internal Medicine, Hanyang University College of Medicine, Hanyang University Seoul Hospital, Seoul, Republic of Korea; International University of Health and Welfare, School of Medicine, JAPAN

## Abstract

**Background:**

The association between renal function and all-cause mortality in patients with hypertensive crisis remains unclear. We aimed to identify the impact of estimated glomerular filtration rate (eGFR) on all-cause mortality in patients with hypertensive crisis visiting the emergency department (ED).

**Methods:**

This retrospective study included patients aged ≥18 years admitted to the ED between 2016 and 2019 for hypertensive crisis (systolic blood pressure ≥180 mmHg and/or diastolic blood pressure ≥110 mmHg). They were classified into four groups according to the eGFR at admission to the ED: ≥90, 60–89, 30–59, and <30 mL/min/1.73 m^2^.

**Results:**

Among the 4,821 patients, 46.7% and 5.8% had an eGFR of ≥90 and <30 mL/min/1.73 m^2^, respectively. Patients with lower eGFR were older and more likely to have comorbidities. The 3-year all-cause mortality rates were 7.7% and 41.9% in those with an eGFR ≥90 and <30 mL/min/1.73 m^2^, respectively. After adjusting for confounding variables, those with an eGFR of 30–59 (hazard ratio [HR], 1.93; 95% confidence interval [CI], 1.47–2.54) and <30 mL/min/1.73 m^2^ (HR, 2.35; 95% CI, 1.71–3.24) had significantly higher 3-year all-cause mortality risks than those with an eGFR of ≥90 mL/min/1.73 m^2^. Patients with an eGFR of 60–89 mL/min/1.73 m^2^ had a higher mortality (21.1%) than those with an eGFR of ≥90 mL/min/1.73 m^2^ (7.7%); however, the difference was not significant (HR, 1.21; 95% CI, 0.94–1.56).

**Conclusions:**

Renal impairment is common in patients with hypertensive crisis who visit the ED. A strong independent association was observed between decreased eGFR and all-cause mortality in these patients. eGFR provides useful prognostic information and permits the early identification of patients with hypertensive crisis with an increased mortality risk. Intensive treatment and follow-up strategies are needed for patients with a decreased eGFR who visit the ED.

## Introduction

Hypertensive crisis is defined as a sudden and abrupt elevation in blood pressure, which may manifest as either a hypertensive emergency or an urgency depending on acute target organ damage, including cardiac, renal, and neurologic injuries [1]. Previous studies have demonstrated that patients with hypertensive crisis have a high mortality rate, and the most common causes of mortality were renal failure (39.7%), stroke (23.8%), myocardial infarction (11.1%), and heart failure (10.3%) [2–5]. Chronic kidney disease (CKD) is a risk factor for hypertensive emergency, and the presence of acute kidney injury (AKI) is associated with a short-term risk of morbidity and mortality in patients with hypertensive crisis [6]. However, it might be difficult to distinguish between AKI and CKD in patients with hypertensive crisis because patients who visit the emergency department (ED) usually have poor baseline renal function, and the percentage changes in the serum creatinine level after AKI are dependent on the baseline renal function [7]. Glomerular filtration rate (GFR) is widely accepted as the best indicator of renal function. It cannot be measured directly but can be estimated from age, sex, race, and serum levels of endogenous filtration markers, such as creatinine and cystatin C [8]. Currently, the estimated glomerular filtration rate (eGFR) is recommended for initial evaluation of renal function in medical practice [9].

Renal dysfunction is associated with mortality and is an independent predictor of cardiovascular events in patients with hypertension, which shows the same predictive value in patients with and without CKD [10–12]. However, there are limited data on the long-term mortality risk associated with renal function in patients with hypertensive crisis. Accurate knowledge of the mortality risk associated with renal function among patients who present with hypertensive crisis could help guide decision-making and risk-reduction efforts among clinicians. Therefore, the aim of this study was to evaluate the potential impact of eGFR on long-term mortality in patients with hypertensive crisis who visited the ED.

## Methods

### Study design and settings

This retrospective study was conducted at a single regional emergency medical center affiliated with a university hospital. Data were collected from patients who visited the ED between January 2016 and December 2019. The study was reviewed and approved by the Institutional Review Board of Hanyang University Guri Hospital and was conducted in accordance with the Declaration of Helsinki. The institutional review board waived the requirement for written informed consent.

### Study population

The study design and detailed descriptions of acute hypertension-mediated organ damage (HMOD) and comorbidities in our study have been previously published [3, 13]. Briefly, this study included patients aged ≥18 years who presented to the ED with hypertensive crisis. Hypertensive crisis was defined as a systolic blood pressure (SBP) ≥180 mmHg and/or a diastolic blood pressure (DBP) ≥110 mmHg [14]. Hypertensive crises were further classified as hypertensive emergencies or hypertensive urgencies according to the presence of acute HMOD. Patients with acute trauma or those who only needed a medical certificate were excluded; when they visited the ED multiple times, only the data from the first visit were included. Patients who did not have a serum eGFR measurement at the index ED visit and those receiving regular dialysis were excluded.

### Data collection and outcomes

After enrollment, additional data were collected from electronic medical records by trained data collectors under the supervision of the principal investigator. The collected data included clinical characteristics, cardiovascular risk factors, comorbidities, presence of acute HMOD, diagnostic test findings, and events during hospitalization or follow-up (e.g., admission, readmission, discharge, and ED revisits). Data on the timing and overall incidence of mortality were extracted from the National Health Insurance Service of the Republic of Korea. Enrolled patients were followed up until death from any cause or until the end of the study (March 2021).

### Definitions of renal function

**Fig 1:**
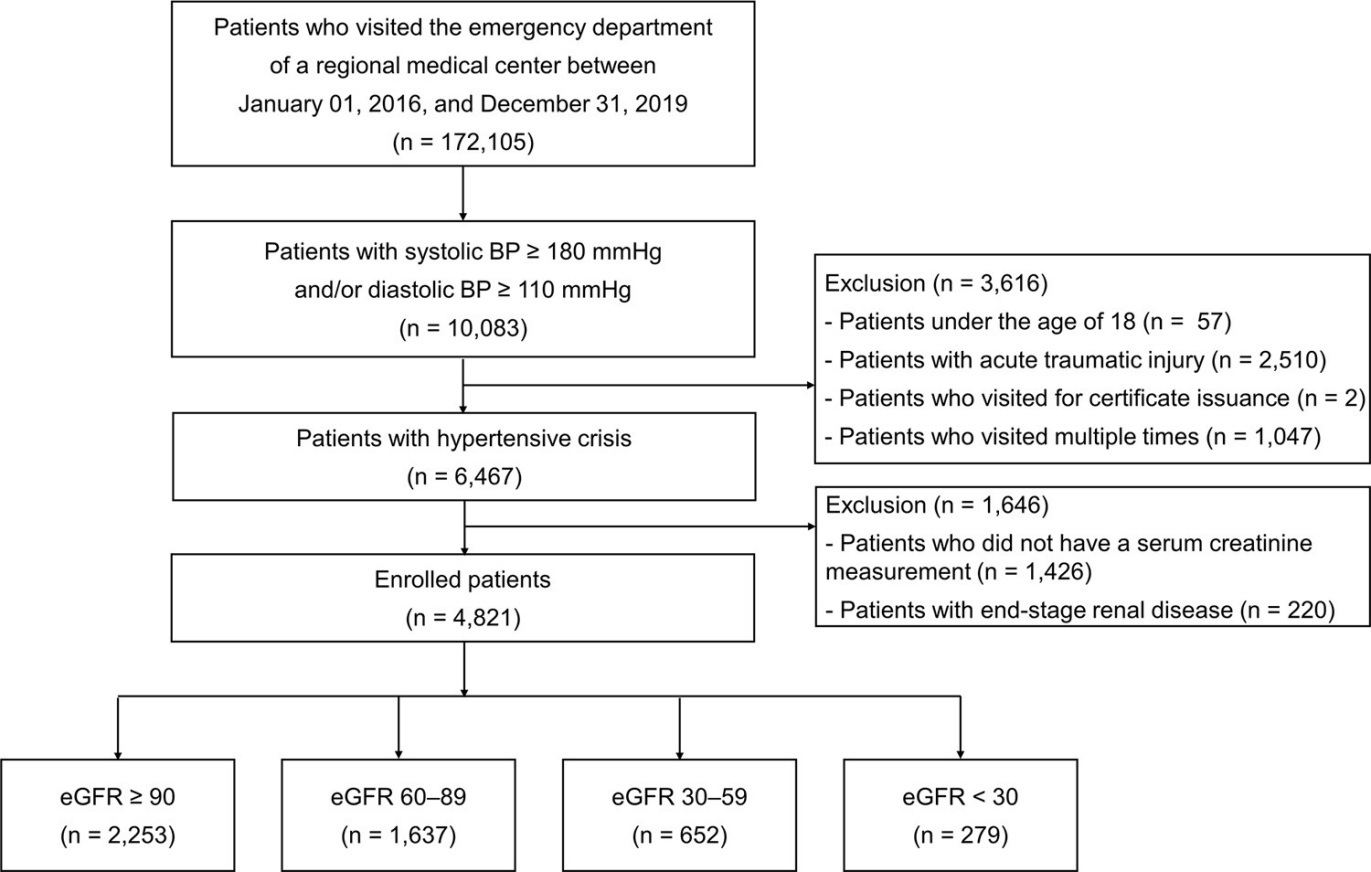
Study flowchart.

eGFR was estimated using the equation derived from the CKD Epidemiology Collaboration equation [15]: The patients were classified according to the eGFR at initial presentation, per clinical practice guidelines [16]: ≥90 mL/min/1.73 m^2^, 60–89 mL/min/1.73 m^2^, 30–59 mL/min/1.73 m^2^, and <30 mL/min/1.73 m^2^ (Fig 1).

### Statistical analysis

Continuous variables are presented as means (standard deviations) or medians (interquartile ranges). Categorical variables are summarized as frequencies (percentages). Univariate linear regression was performed to test for linear trends among continuous variables. The Mantel–Haenszel χ^2^ test was used to compare categorical variables. The 3-year all-cause mortality was estimated using Kaplan–Meier survival analysis, and group comparisons were performed using the log-rank test. The independent predictive value of renal dysfunction for 3-year all-cause mortality was determined using a Cox proportional hazard regression model, with consideration of other clinically relevant variables, including baseline characteristics (age, sex, SBP, and DBP), comorbidities (hypertension, diabetes mellitus, dyslipidemia, ischemic stroke, hemorrhagic stroke, coronary artery disease, heart failure, and peripheral artery disease), components of subclinical HMOD (proteinuria, cardiomegaly on chest radiography, and left ventricular hypertrophy [LVH], myocardial ischemia and atrial fibrillation on electrocardiography [ECG] findings). Subgroup analyses were performed by controlling for covariates. Hazard ratios (HRs) and the corresponding 95% confidence intervals (CIs) were calculated. All tests were two-tailed, and statistical significance was set at *p*<0.05. All analyses were performed using Statistical Analysis Software package (version 9.4; SAS Institute, Cary, NC, USA).

## Results

### Baseline characteristics

A total of 4,821 patients were enrolled in the study, and follow-up data for up to 5.2 years were analyzed. The median follow-up period was 2.8 years (interquartile range, 1.8–3.9 years). Among the enrolled patients, 53.3% had a decreased renal function defined as an eGFR of <90 mL/min/ 1.73 m^2^; 34.0% (n = 1,637) had an eGFR of 60–89 mL/min/1.73 m^2^; 13.5% (n = 652) had an eGFR of 30–59 mL/min/1.73 m^2^; and 5.8% (n = 279) had an eGFR of <30 mL/min/1.73 m^2^. Table 1 displays the baseline characteristics according to eGFR. In general, patients with a lower eGFR were older (*p*<0.001) and had higher SBP (*p*<0.001) than their counterparts. The prevalence of hypertension, diabetes mellitus, dyslipidemia, ischemic stroke, hemorrhagic stroke, coronary artery disease, peripheral artery disease, and heart failure tended to increase with decreasing eGFR. In addition, patients with a lower eGFR showed higher serum creatinine, troponin-I, brain natriuretic peptide, and D-dimer levels, and a lower hemoglobin level than their counterparts. The incidence of proteinuria, cardiomegaly on chest radiography, and LVH on ECG showed similar trends that increased with declining eGFR (all *p*<0.001, except for the troponin-I level [*p* = 0.016] and LVH on ECG [*p* = 0.008]). Acute HMOD was more frequently observed in patients with a lower eGFR than in their counterparts (*p*<0.001).

**Table 1:** Baseline characteristics.

	All patients (n = 4,821)	eGFR (mL/min/1.73 m^2^)				*p*-value for trend
		≥90 (n = 2,253)	60–89 (n = 1,637)	30–59 (n = 652)	<30 (n = 279)	
Age, years, mean (SD)	62.7 (16.9)	52.3 (13.9)	70.4 (13.6)	75.7 (12.7)	70.6 (14.8)	<0.001
Female sex, n (%)	2,345 (48.6)	1,059 (47.0)	789 (48.2)	348 (53.4)	149 (53.4)	0.002
Medical history, n (%)						
Hypertension	2,532 (53.9)	834 (38.2)	991 (62.4)	487 (75.5)	220 (79.4)	<0.001
Diabetes mellitus	1,241 (26.7)	337 (15.6)	452 (28.7)	285 (44.7)	167 (60.3)	<0.001
Dyslipidemia	469 (10.2)	187 (8.7)	161 (10.3)	83 (13.1)	38 (14.0)	<0.001
Ischemic stroke	400 (8.7)	89 (4.2)	183 (11.7)	86 (13.5)	42 (15.4)	<0.001
Hemorrhagic stroke	128 (2.8)	27 (1.3)	58 (3.7)	33 (5.2)	10 (3.4)	<0.001
Coronary artery disease	442 (9.6)	103 (4.8)	196 (12.5)	106 (16.7)	37 (13.6)	<0.001
Peripheral artery disease	37 (0.8)	8 (0.4)	18 (1.2)	5 (0.8)	6 (2.2)	0.002
Heart failure	190 (4.1)	17 (0.8)	65 (4.2)	68 (10.8)	40 (14.6)	<0.001
Social history, n (%)						
Cigarette smoking	840 (26.1)	519 (37.3)	193 (17.0)	84 (17.9)	44 (19.7)	<0.001
Alcohol consumption	1,183 (36.2)	711 (49.3)	339 (30.0)	92 (19.5)	41 (18.4)	<0.001
Triage vital signs, mean (SD)						
SBP, mmHg	190.3 (22.1)	187.3 (21.6)	192.3 (21.1)	192.5 (23.3)	197.1 (25.7)	<0.001
DBP, mmHg	107.8 (17.5)	111.6 (15.2)	105.1 (18.1)	104.1 (19.3)	102.7 (20.8)	<0.001
Laboratory test results						
Serum creatinine, mg/dL (SD)	1.06 (1.15)	0.70 (0.15)	0.89 (0.21)	1.32 (0.30)	4.31 (3.18)	<0.001
Troponin-I level, ng/mL (SD)	0.17 (2.35)	0.11 (1.12)	0.17 (2.57)	0.24 (1.99)	0.50 (5.60)	0.016
BNP level, pg/mL (SD)	295 (628)	101 (241)	245 (479)	467 (703)	971 (1293)	<0.001
D-dimer level, mg/L (SD)	778 (2922)	337 (1031)	872 (3513)	1256 (3049)	1659 (5178)	<0.001
Hb, level g/dL (SD)	13.6 (2.1)	14.2 (1.7)	13.7 (2.0)	12.6 (2.2)	10.8 (2.5)	<0.001
Urinary analysis, n (%)	3,300 (68.5)	1,415 (62.8)	1,145 (69.9)	515 (79.0)	225 (80.6)	<0.001
Proteinuria^a^, n (%)	1,073 (32.5)	261 (18.4)	353 (30.8)	273 (53.0)	186 (82.7)	<0.001
Chest radiography, n (%)	4,522 (93.8)	2,098 (93.1)	1,537 (93.9)	618 (94.8)	269 (96.4)	0.015
Cardiomegaly, n (%)	617 (13.6)	180 (8.6)	259 (16.8)	117 (18.9)	61 (22.8)	<0.001
Congestion, n (%)	396 (8.7)	146 (6.9)	150 (9.7)	57 (9.2)	43 (16.0)	<0.001
ECG, n (%)	4,216 (87.5)	1,876 (83.3)	1,471 (89.9)	599 (91.9)	270 (96.8)	<0.001
LVH, n (%)	498 (11.8)	183 (9.8)	204 (13.9)	76 (12.7)	35 (13.0)	0.008
Myocardial ischemia, n (%)	287 (6.8)	110 (5.9)	109 (7.4)	50 (8.4)	18 (6.7)	0.078
Atrial fibrillation, n (%)	244 (5.8)	60 (3.2)	111 (7.6)	53 (8.9)	20 (7.4)	<0.001
Acute HMOD, n (%)	1,686 (35.0)	566 (25.1)	618 (37.8)	329 (50.5)	173 (62.0)	<0.001

Data are presented as n (%) or mean (SDs), as appropriate. SD, standard deviation; eGFR, estimated glomerular filtration rate; SBP, systolic blood pressure; DBP, diastolic blood pressure; BNP, B-type natriuretic peptide; Hb, hemoglobin; ECG, electrocardiography; LVH, left ventricular hypertrophy; HMOD, hypertension-mediated organ damage.

^a^Proteinuria was defined as a dipstick urinalysis result of ≥1+.

### Outcomes during the index visit and follow-up

A total of 2,548 (52.9%) patients were directly admitted to the hospital ward, 1,786 (37.1%) patients were discharged, 482 (10.0%) were discharged against medical advice, and 5 (0.1%) died in the ED. At the index ED visit, the admission rate increased with a decrease in eGFR (*p*<0.001). The overall ED revisit rates within 1 month, 3 months, and 1 year were 9.4%, 16.4%, and 28.3%, respectively. The overall readmission rates within 1 month, 3 months, and 1 year were 6.3%, 8.9%, and 13.5%, respectively. There were no significant differences in the ED revisit and readmission rates within 1 month according to the eGFR; however, the ED revisit and readmission rates within 3 months and 1 year were higher in the patients with a lower eGFR than in their counterparts (Table 2).

**Table 2:** Outcomes during the index visit to the ED and follow-up.

	All patients (n = 4,821)	eGFR (mL/min/1.73 m^2^)				*p*-value for trend
		≥90 (n = 2,253)	60–89 (n = 1,637)	30–59 (n = 652)	<30 (n = 279)	
Outcomes of the index visit to the ED, n (%)						
Admission	2,548 (52.9)	993 (44.1)	913 (55.8)	433 (66.4)	209 (74.9)	<0.001
Discharge	1,786 (37.1)	1,043 (46.3)	560 (34.2)	146 (22.4)	37 (13.3)	<0.001
Discharge against medical advice	482 (10.0)	217 (9.6)	163 (10.0)	72 (11.0)	30 (10.8)	0.297
Death in the ED	5 (0.1)	0 (0)	1 (0.1)	1 (0.2)	3 (1.1)	<0.001
Revisit to the ED, n (%)						
Within 1 month	360 (9.4)	158 (9.1)	124 (9.4)	55 (10.3)	23 (9.8)	0.476
Within 3 months	628 (16.4)	265 (15.3)	212 (16.1)	91 (17.0)	60 (25.5)	0.001
Within 1 year	1,083 (28.3)	447 (25.8)	370 (28.0)	178 (33.2)	88 (37.5)	<0.001
Readmission, n (%)						
Within 1 month	241 (6.3)	100 (5.8)	90 (6.8)	35 (6.5)	16 (6.8)	0.342
Within 3 months	342 (8.9)	135 (7.8)	114 (8.6)	59 (11.0)	34 (14.5)	0.003
Within 1 year	516 (13.5)	198 (11.4)	177 (13.4)	88 (16.4)	53 (22.6)	<0.001
Mortality, n (%)						
Within 1 month	195 (4.0)	35 (1.6)	70 (4.3)	57 (8.7)	33 (11.8)	<0.001
Within 3 months	298 (6.2)	59 (2.6)	113 (6.9)	81 (12.4)	45 (16.1)	<0.001
Within 1 year	568 (11.8)	113 (5.0)	212 (13.0)	163 (25.0)	80 (28.7)	<0.001

Data are presented as n (%). ED, emergency department. eGFR, estimated glomerular filtration rate.

### All-cause mortality during follow-up

**Fig 2:**
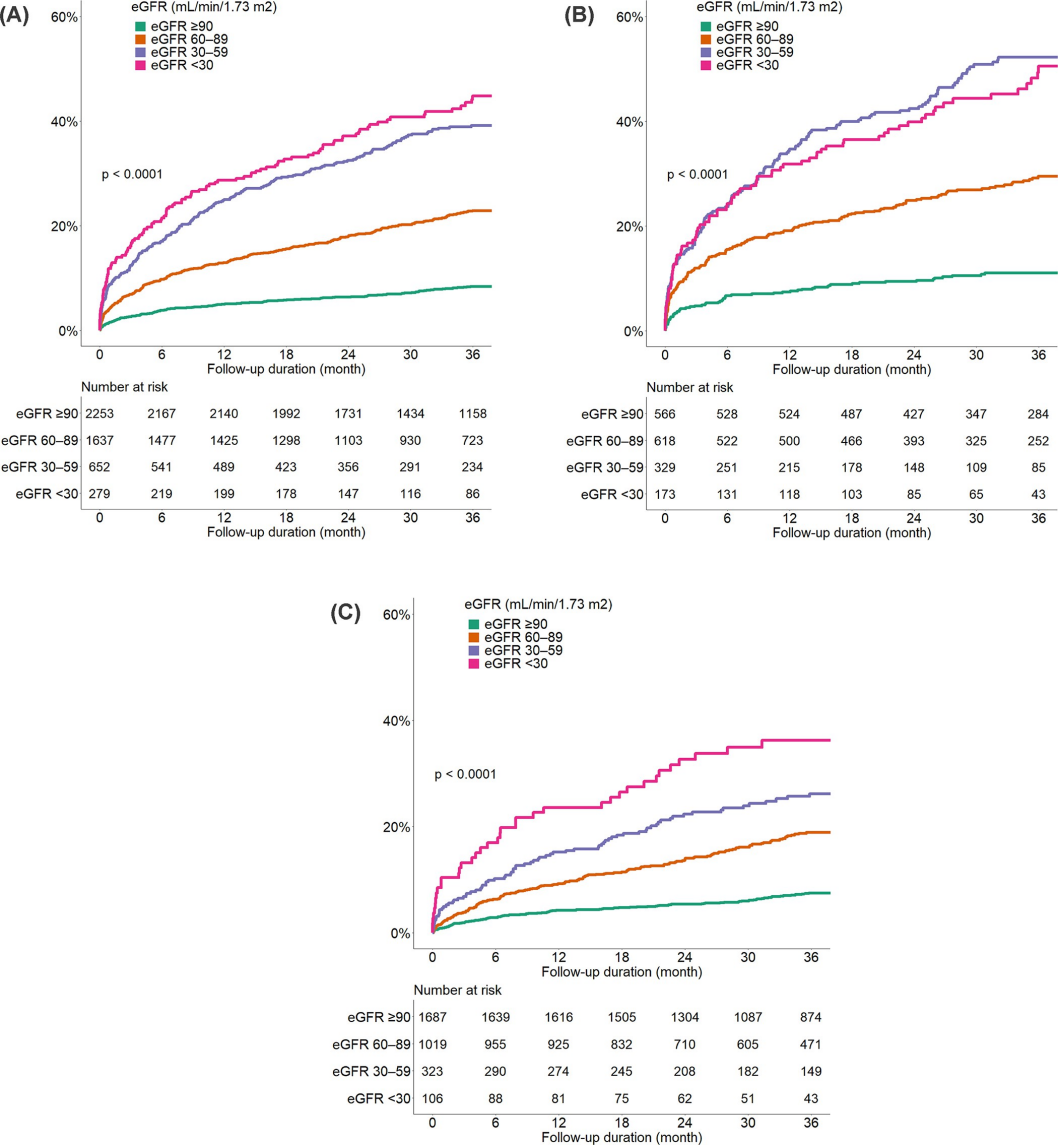
Cumulative all-cause mortality according to the estimated glomerular filtration rate in (A) all patients, (B) patients with hypertensive emergency, and (C) patients with hypertensive urgency.

During follow-up, the unadjusted 3-year all-cause mortality increased with a decline in eGFR in all patients (Fig 2A). The 3-year all-cause mortality was 7.7% in patients with an eGFR of ≥90 mL/min/1.73 m^2^ (lowest risk) and 41.9% in patients with an eGFR of <30 mL/min/1.73 m^2^ (highest risk). Similar trends were observed in the subgroups with acute HMOD (Fig 2B) and without acute HMOD (Fig 2C). Among the subgroups with hypertensive emergency and hypertensive urgency, the 3-year all-cause mortality was 10.6% and 6.7% in patients with an eGFR of ≥90 mL/min/1.73 m^2^ and 46.2% and 34.9% in those with an eGFR of <30 mL/min/1.73 m^2^, respectively (Table 3).

**Table 3:** All-cause mortality and hazard ratios for mortality according to the eGFR among the patients with hypertensive crisis.

eGFR (mL/min/1.73 m^2^)	All patients (n = 4,821)			Patients with hypertensive emergency (n = 1,686)			Patients with hypertensive urgency (n = 3,135)		
	3-year mortality	HR (95% CI)	aHR^a^ (95% CI)	3-year mortality	HR (95% CI)	aHR^a^ (95% CI)	3-year mortality	HR (95% CI)	aHR^a^ (95% CI)
≥90	173 (7.7)	REF	REF	60 (10.6)	REF	REF	113 (6.7)	REF	REF
60–89	345 (21.1)	2.95 (2.46–3.54)	1.21 (0.94–1.56)	172 (27.8)	2.90 (2.16–3.89)	1.38 (0.94–2.02)	173 (17.0)	2.67 (2.11–3.39)	1.04 (0.74–1.47)
30–59	240 (36.8)	5.75 (4.73–6.99)	1.93 (1.47–2.54)	161 (48.9)	5.92 (4.40–7.97)	2.43 (1.63–3.62)	79 (24.5)	4.03 (3.02–5.37)	1.16 (0.77–1.74)
<30	117 (41.9)	6.81 (5.39–8.61)	2.35 (1.71–3.24)	80 (46.2)	5.46 (3.90–7.63)	2.42 (1.56–3.76)	37 (34.9)	6.22 (4.29–9.02)	1.93 (1.11–3.34)

Data are presented as n (%). HR, hazard ratio; aHR, adjusted hazard ratio; CI, confidence interval; eGFR, estimated glomerular filtration rate.

^a^Adjusted for age, sex, systolic blood pressure, diastolic blood pressure, proteinuria, cardiomegaly on chest radiography, left ventricular hypertrophy, myocardial ischemia, and atrial fibrillation on electrocardiography, and medical history of hypertension, diabetes mellitus, dyslipidemia, ischemic stroke, hemorrhagic stroke, coronary artery disease, heart failure, and peripheral artery disease.

After adjustments for age, sex, SBP, DBP, comorbidities, components of subclinical HMOD, and ECG findings in the multivariable Cox regression analysis among all patients, those with an eGFR of 30–59 mL/min/1.73 m^2^ (adjusted HR, 1.93; 95% CI, 1.47–2.54) and <30 mL/min/1.73 m^2^ (adjusted HR, 2.35; 95% CI, 1.71–3.24) showed a significantly higher risk of 3-year all-cause mortality than those with an eGFR of ≥90 mL/min/1.73 m^2^. Among the subgroup with hypertensive emergency, the patients with an eGFR of 30–59 mL/min/1.73 m^2^ (adjusted HR, 2.43; 95% CI, 1.63–3.62) and <30 mL/min/1.73 m^2^ (adjusted HR, 2.42; 95% CI, 1.56–3.76) had an increased risk of 3-year all-cause mortality; among the subgroup with hypertensive urgency, only the patients with an eGFR of <30 mL/min/1.73 m^2^ showed a significantly increased risk of 3-year all-cause mortality (adjusted HR, 1.93; 95% CI, 1.11–3.34) (Table 3).

## Discussion

Our study showed that a decreased eGFR was an independent risk factor for mortality within 3 years in patients with hypertensive crisis. Moreover, the association between decreased eGFR and increased risk of all-cause mortality persisted even after adjusting for confounding factors, such as age, comorbidities, and subclinical HMOD. Similar trends were observed in the subgroups according to the presence or absence of acute HMOD; these trends were more prominent in patients with hypertensive emergency than in those with hypertensive urgency.

Renal dysfunction, defined as an eGFR of <60 mL/min/1.73 m^2^, is well recognized as a strong predictor of future adverse cardiovascular outcomes, including mortality, in the general population; its predictive ability is even higher among high-risk patients with diabetes and hypertension than among the general population [17]. Moreover, it is a risk factor for mortality among patients with other forms of cardiovascular disease, such as acute myocardial infarction [18] and heart failure [19]. However, there is limited knowledge regarding the association between renal dysfunction and all-cause mortality in patients with hypertensive crisis. Szczech et al. reported that among patients admitted to the hospital for acute severe hypertension, the presence of either CKD or AKI was associated with worse outcomes, including an increased length of hospitalization; furthermore, AKI was associated with 90-day mortality, but not CKD [6]. Our study demonstrated a strong association between decreased eGFR and long-term mortality, as well as the ED revisit and readmission rates in patients with hypertensive crisis. Patients with an eGFR of <30 mL/min/1.73 m^2^ showed a remarkably high mortality of 28.7% within 1 year and 41.9% within 3 years. These rates are close to those reported in patients with renal insufficiency treated for heart failure or acute myocardial infarction. Analysis of the data from 22 heart failure studies included in the Meta-Analysis Global Group in Chronic Heart Failure showed an all-cause mortality of 45.6% over a median follow-up period of 2 years in 1,705 patients with heart failure and an eGFR of <30 mL/min/1.73 m^2^ [20]. In a study of Koreans with myocardial infarction, 1-year composite major adverse cardiac events occurred more frequently in patients with an eGFR <30 mL/min/1.73 m^2^ (31.5%) than in those with an eGFR of >60 mL/min/1.73 m^2^ (9.4%) [21].

In this study, an increased risk of all-cause mortality was observed in patients with hypertensive crisis who had an eGFR of 30–59 mL/min/1.73 m^2^ and <30 mL/min/1.73 m^2^. There are a few possible explanations for these findings. First, patients with decreased eGFR had a higher incidence of acute HMOD than their counterparts. Second, patients with a decreased eGFR had more established cardiovascular diseases, such as ischemic stroke, coronary artery disease, and heart failure, and risk factors, such as diabetes mellitus and dyslipidemia, than their counterparts. Third, patients with a decreased eGFR more frequently showed findings of subclinical HMOD, such as proteinuria, cardiomegaly on chest radiography, and LVH on ECG [22–24], and had higher BNP, D-dimer, and troponin-I levels and lower hemoglobin levels, which are associated with a risk of cardiovascular morbidity and mortality, than their counterparts [25–27].

Moreover, the association between decreased eGFR and increased risk of all-cause mortality persisted even after adjusting for confounding factors, such as age, comorbidities, and subclinical HMOD. There are several possible reasons for this finding. Patients with a decreased eGFR may have CKD or AKI superimposed on CKD. CKD is a direct cause of mortality and a risk factor for cardiovascular disease, and patients with hypertension and CKD have a high cardiovascular risk [28]. In addition, a decreased eGFR is not only representative of renal dysfunction but is also associated with various pathological conditions, such as inflammation, microvascular dysfunction, and enhanced oxidative stress, which share a common pathway in the development of atherosclerosis and heart failure [29–31]. In this regard, clinical guidelines recommend screening for CKD in individuals with hypertension and more intensive interventions in patients with CKD to prevent adverse outcomes [14]. Our study highlights the importance of evaluating CKD, even in patients with highly specific hypertension with acute and severe increases in blood pressure.

Another impressive finding of our study was that the correlation between decreased eGFR and all-cause mortality was more prominent in patients with hypertensive emergency than in those with hypertensive urgency. This could be the result of the interaction between renal dysfunction and damage to other organs, which are frequently observed in patients with hypertensive emergencies. It is well understood that cardiorenal syndrome reflects conditions in which failure of either the heart or the kidney leads to or accelerates the failure of other organs [32]. In addition, the prevalence of multimorbidity is increasing rapidly, and any combination of multimorbidities, such as diabetes mellitus, stroke, myocardial infarction, and CKD, is associated with a multiplicative mortality risk [33, 34]. In this respect, the impact of a decreased eGFR may be more prominent in patients with hypertensive emergency who are older and have more comorbidities [3] than in those with hypertensive urgency. However, it is noticeable that an eGFR of <30 mL/min/1.73 m^2^ was also a significant poor prognostic factor even in patients with hypertensive urgency, usually considered to be relatively low-risk patients by clinicians.

Taken together, our findings showed that early risk stratification is important to identify patients at a higher risk for mortality and that more intensive treatment and follow-up strategies are needed for patients with hypertensive crisis and renal dysfunction visiting the ED. In this respect, routine assessment of renal function, especially eGFR, to rule out asymptomatic renal injuries and prognostic risk stratification would have clinical implications. Future research regarding eGFR for predicting cardiovascular and renal events or mortality in patients with hypertensive crisis is needed.

Our study had several limitations. First, given the nature of this study, retrospective data on the baseline characteristics of the study population were insufficient compared to the data used in prospective studies. In addition, we could not obtain data on the reasons for revisit or readmission after the index ED visits. Second, it could not be confirmed that the observed causal relationships excluded all confounding factors. Third, the study included data from a single center, which may not be representative of the entire Korean population. Fourth, we used only baseline eGFR values and did not consider the follow-up eGFR results. Finally, we could not identify cardiovascular and renal events or cardiovascular mortality in this study because the National Health Insurance Service did not provide information on the cause of mortality. However, the data regarding all-cause mortality and the date of mortality were highly accurate because they were obtained from the National Health Insurance Service, which covers the entire Korean population. Further prospective studies are needed to determine the optimal screening, risk stratification and treatment strategies related to cardiovascular events or deaths according to eGFR levels in patients with hypertensive crisis.

## Conclusions

Decreased eGFR was a strong independent predictor of mortality in patients with hypertensive crisis. The mortality increased with a declining eGFR, and the impact of a decreased eGFR on all-cause mortality was more pronounced in the patients with hypertensive emergency than in the patients with hypertensive urgency. Effective screening based on eGFR, early identification of increased risks of mortality, appropriate treatment, and close follow-up strategies are essential to improve the outcomes of patients with hypertensive crisis visiting the ED.
